# Dental Education

**Published:** 1884-08

**Authors:** A. Jackson


### THE DENTAL REGISTER.

Vol. XXXVIII.] AUGUST, 1884. [No. 8.

### Communications.

### Dental Education.

BY A. JACKSON.

Read before the Alabama Dental Society.

Artemus Ward, better known to some as "Old Waxworks," conceived the idea of killing his rival by reading his lectures to him, and intimated that he would expire before he had half finished reading them. This idea suggests a valuable expedient to me in the present crisis. As my name did not appear in the published proceedings of the Convention of last year, I concluded that Dr. *Manifest Destiny*, or some irrepressible reformer, had improved upon the wisdom of the Convention by leaving me out of the programme for the present occasion. This impression was not removed until it was too late for me to make the careful preparation which my subject demanded. Feeling reluctant to appear before such a talented body without adequate preparation, I was at first strongly inclined merely to state the untoward circumstances, and say to you, in the language of Scripture, "I pray thee have me excused;" but, tempted by the wicked suggestion of Ward, I thought that I might get rid of some of my dangerous rivals in the profession by torturing them to death with a *poor* paper, and, with an eye to the main chance, I then decided to embrace the opportunity.

The genius of Chaucer furnishes a beautiful allegory, that may be employed to lead the way up to my subject. The poet's fancy transports him beyond the moon and the stars, and puts him down before the Temple of Fame. This grand edifice, built of emerald, with shining towers and gorgeous columns, rests upon an immense iceberg, which is high and difficult to climb. The southern side of the iceberg was inscribed with the names of men who had been famous in art, science, and literature, but being exposed to the heat of the sun they were constantly melted away. On the northern side, where they were protected against the heat of the sun, the names remained legible and permanently graven. The poet enters the temple, and a gorgeous and magnificent scene greets his eyes. In a spacious apartment, lined with gold and studded with pearls, on a glittering throne, sits the Queen of the Temple. Around her stand the pillars, upon which are incribed the greatest names known to art, literature, and science—those who climbed over the iceberg, and left their names where they would not be melted away. The allegory of the poet teaches us an important lesson. It teaches that the road to learning is not over level plains nor smooth Appian ways, but over icy cliffs and rugged promontories. To secure the acquisition of knowledge, there must be effort, and the more profound the knowledge, the greater must be the effort.

Skilled brain power and the treasures of a well-stored intellect are not the heritage of man. He has the power to think, to reason, to master difficult problems, to search into the secrets of nature, and to penetrate the mysteries of science with his keen intellect; but all these require thought, research, and unremitting effort. The practical application of this doctrine enabled an obscure Corsican to rise, like a giant from his lethargy, and seat himself on the throne of the Bourbons; it enabled Lord Nelson to decree at Trafalgar that proud England should still be mistress of the seas; and it enabled Cæsar to make Rome so powerful she could sit on her seven hills of beauty and rule the world. It enabled Michael Angelo to carve the beautiful form of an angel out of unsightly rock; Raphael to spread upon canvas his immortal Madonna; and Leonardo to signalize himself as the genius who could grasp art and science with hitherto unknown power. It enabled Virgil to tune his lyre to the praise of Æneas in the sweetest melody; Homer to sing of the exploits of his countrymen in immortal song; and Milton to portray in sublimest verse the daring sacrilege of the fallen angels. It enabled Humboldt to advance to the foremost rank of the world's great scientists; Agassiz to tell, by examining a sea-shell, the age in which the animal lived that inhabited it; and Cyrus W. Field to *perfect* the magnetic telegraph—that wonderful agent by which a man sits at his table in New York, and talks with another who is far beyond the broad Atlantic. The epitome of wonders is not yet exhausted. It has given to chemistry the power to seize great secrets from the laboratory of nature; to engineering the skill to cut railroads through rocky mountains and under deep streams, to build suspension bridges over broad rivers, and to connect remote seas by artificial channels; and it has given to medical science the remedies to heal malignant disease and mitigate human suffering.

Skilled brain power, combined with diligent research and concentrated effort, has achieved marvellous results in our profession during its brief existence. While it is but yesterday since it sprang into being, yet the discoveries it has brought to light, and the mechanical contrivances and remedial agents it has summoned to its aid, justly entitle it to claim equal rank with any of the learned professions. In speaking of its brief existence, we do not wish to be understood as disputing the facts of history bearing upon its origin. Dentistry, in some form, probably existed as far back as the mythic period of Greece. Upon no other hypothesis can we account for the fact that the old classic poets, Ovid and Horace, speak of it. I may add, too, that mummies have been found in Egypt, and skeletons have been taken from the tombs of the ancients, which showed the handicraft of the dentist. In this instance, we are not led to exclaim, "hark! from the tombs a doleful sound,"—but hark! from the tombs a *significant* sound. The conclusion to which these facts point, is, that some attention was paid to it in the earlier ages of the world, but what was known of it as a science perished beneath the torrent of Goth and Vandal ignorance, or went down in the night of time. Its secrets were left buried for a long period, and not until the present century were they brought to light again. John Hunter contributed much to this result by his learned treatises, and thus laid the foundation of the Dental School of England.

The French dentists made wonderful progress in the profession about the same time, and their scientific researches have brought valuable aids to its development. But to Leonard Kroecker, of our own country, is due the credit of causing it to take rank as a distinct profession. He practiced dentistry in New York soon after the revolutionary war, and his scientific lore and mature experience caused it to shoot forth, like a meteor, in a blaze of splendor. Since then it has always ranked as a separate profession. It now stands upon an equal footing with that of the aurist, the oculist, and the medical practitioner. Its claims to scientific recognition have been placed beyond all question, by the schools of learning it has established and the valuable discoveries it has given to suffering humanity. Science is the keystone upon which it rests. It calls to its aid anatomy, philosophy, physiology, mineralogy, the secrets of chemistry, and the principles of mechanism. These great agencies are the talismanic sounds that have lifted it into an elevated position in the minds of the intelligent and educated, and to them must we look to sustain its well-earned prestige. The rise and progress of dental science, its aims and results, are interesting, not only to members of the profession, but to all persons of inquiring minds and liberal culture. During our war of Independence, there was only one dentist on the Western Continent, and he was one of the noble Frenchmen who came to the rescue of the colonies in the most critical hour of their fortunes. When George Washington was President of the United States, there was but one in the great city of New York, and to his honor be it said, he prepared a full set of ivory teeth for "the Father of his Country." When Alabama was admitted into the Union, there were only one hundred in all the States. Now the aggregate number reaches far up into the thousands, and they are found in almost every city, town, and hamlet of the thickly populated portions of the country. This statistical exhibit is pregnant with meaning. It shows the level of the times, and that, so far as public favor is concerned, we are fully abreast of our sister professions in this age of progress and scientific lore.

Much of our progress has been due to our schools and colleges and to our State and national conventions of dentists, by means of which we are educated in the latest discoveries of science. Another great cause of our progress results from the fact that our attention is mainly directed to the treatment of a single organ. The chief reason why more is not accomplished, in the learned professions, is because mental power is not concentrated upon special branches. Men achieve but little, because their labors are diversified. The road to success is through specialties. Mental power, like a large stream, wastes its forces when spread over a vast surface, but when concentrated, it sweeps onward with a momentum of a mighty cataract. Our age is too complex for one to master learning and grasp all the sciences, but if he knocks with resolute hand at the door-way of a special branch, he has strong reason to believe that "to him that knocketh, it shall be opened." "The man that seeks but one thing in life, and but one, may hope to achieve it before life be done."

Although the French, English, and American schools of dentistry have done much to acquaint us with the secrets of first dentition and the diseases that accompany it, as well as the different subjects relating to the prevention and cure of the diseases of the teeth; yet the rapid progress for which we are indebted to them, can be better illustrated by referring to the improvements in mechanical dentistry. It appears from the advertisements of old newspapers that this branch of our profession was once in the hands of jewelers and silversmiths, but our handicraft has set at naught this branch of their business as effectually as the mission of the Apostle Paul set at naught the trade of Demetrius, the silversmith, and his fellow-craftsmen.

We shall now notice some of the improvements which our dental schools have bestowed upon the world. The primitive method of fastening teeth by ligatures and flax, has given way to the method of securing them by metallic clasps and spiral springs, and these, in turn, have been supplanted by the excellent method of securing them, in whole or in partial sets, to a plate of gold or vulcanized rubber so accurately fitted to the gums that they are retained by atmospheric pressure. Teeth which were formerly made from the tusk of the elephant and the tooth of the hippopotamus, and which became offensive, and, owing to their susceptibiliy to the taction of the fluids of the mouth, induced disease in sound teeth, have, under the combined skill of the dentist and chemist, been supplanted by teeth made of porcelain, which effectually resist both, and imitate the natural teeth so perfectly, it requires a sharp eye to distinguish them. "Them pullies" which, in the hands of an ignorant ditcher, with no other qualification than the muscular power to wield them, made the extraction of teeth dangerous, have been displaced by superior instruments, by means of which teeth are now extracted with skill and safety by the professional operator.

Among the many ingenious contrivances which modern dentistry has called to its aid, we may mention automatic mallets and drills worked by treadles and galvanism as a motive force, for preparing cavities and filling teeth; also, the process called continuous gum—colored by mineral oxydes to imitate nature—and cheek restorers, which serve to distend the cheeks when they have fallen in. Since the contrivances of modern dentistry have been brought into requisition, there is no longer any necessity for the old maids to replace their lost teeth with wax-work of their own manufacture, for, though one be toothless and hollow-jawed like the old gypsy, Meg Merriles, yet the dental surgeon who is up to his business can supply her with mouth-works which even the beautiful "Jersey Lilly" might well covet. The Eastern Magi, in their wildest flights of imagination, never conceived such wonders as these.

We have alluded to the fact that some of the Egyptian mummies—three thousand years old, perhaps—have artificial teeth fastened on metallic clasps. When Mark Twain was in Genoa, seeing that his guide took great delight in exciting admiration, he resolved to play off a joke on him by maintaining a stupid indifference in the presence of the sublimest wonders that were shown to him. After this, the guide nearly walked his feet off hunting up extraordinary things, and taxed his ingenuity to the utmost to excite interest in him; but it was a failure. The greatest wonder, a royal Egyptian mummy—the best preserved in the world—was reserved to the last; but that, too, was viewed with stolid indifference. If, however, it had been supplied with the best specimens of modern dental work, such as continuous gum, cheek restorers, and porcelain teeth secured on a gold plate so well fitted as to be kept in place by atmospheric pressure, we are fully satisfied the humorist would have lost his self-possession, and gone into ecstacies over the wonderful curiosity.

The importance of dental education becomes more apparent when we consider that its prime object is to relieve pain, and promote the health and happiness of the human race. Owen Meredith tells us we may live without poetry, without music, without conscience, and without books, but we can't live without cooks. To this may be added, we can't live and enjoy health without the teeth to masticate the food the cooks prepare; and without health even the dreams of romance and the splendors of kingly magnificence can not be enjoyed. "O, blessed Health! thou art above all Gold and Treasure." The machinery of man is so delicate, so complicated, and so nicely adjusted, that the disease of a single nerve will throw his whole organism out of order, and thus prevent the parts from performing their functions. It is the province of the dental surgeon to minister to some of the worst of these nerve disorders, and prevent and cure their racking torture. "*Ton odontos to achos*," or toothache in its worst form, is one of the most malignant of these nerve disorders.

The poet Burns, in his "Address to the Toothache," says:

"When fever burns, or ague freezes,
Rheumatics gnaw, or colic squeezes,
Our neighbor's sympathy may ease us
With pitying moan;
But thee, thou hell of all diseases,
Ay mocks our groan!"

We have heard of martyrs being burned at the stake without faltering, of kings and princes who showed heroic fortitude under the executioner's axe, but we have never heard of any one who could bear a severe toothache without showing signs of intense agony. Even the philosophers can not bear it with patience, although they have made a "pish" at pain and suffering, and written in the style of Gods.

Our dental journals, our schools and colleges, and our State and National Conventions are fine media for disseminating knowledge and communicating the improvements in our profession, and they have accomplished much in their respective departments. A high tide of success has left its unmistakable marks, but the moon is not full, and the highest tide has not yet been reached. However incredible it may appear to some, we have, in the researches of our profession, probably been imitating Isis, who hunted and gathered up the limbs of the good Osiris, after they had been hewed into many pieces by the Egyptian Typhon and his fellow-conspirators, and scattered in unknown places. We have gathered up many parts which had, perhaps, been hewed to pieces and scattered by the rude hands of Goth and Vandal when they laid waste Europe, but there are still others that we may collect by careful search and mould into our beautiful system. Many valuable secrets still slumber amid the mysteries of chemistry, and much may yet be done to relieve human suffering. In a better way we may yet learn how to stop the flow of blood, how to reduce inflammations, how to remove purulent matter, and how to repair and correct the deformities and distributions of nature.

Ours is a noble and honorable profession, and our mission is one of mercy and humanity. It behooves us to work earnestly and zealously to raise it still higher on the pillars of science, and to elevate its ethical and humanitarian standard. There may be no temples erected to pay us Divine honors, such as were paid to Æsculapius, the legendary father of surgery; there may be no Homer to immortalize us with such songs as he sung of Machaon, the surgeon who accompanied Agamemnon in the Trojan war, and ministered to the wounded Grecian heroes; but let us remember that there is an aristocracy of talent, and

> "Who e'er amidst the sons
> Of 'Science,' Valor, Liberty, and Virtue,
> Displays distinguished Merit, is a Noble
> Of Nature's own creating."

Taking as our motto the fine sentiment of Terence, "I am a man, and all things human touch me," let us press onward and upward, and, with compassion in our hearts, make it the thought, the wish, and the dream of our lives to help and to heal the sick who lean on us in their afflictions.

